# A survey of attitudes toward clinical research among physicians at Kyoto University Hospital

**DOI:** 10.1186/1472-6920-9-75

**Published:** 2009-12-22

**Authors:** Eriko Sumi, Toshinori Murayama, Masayuki Yokode

**Affiliations:** 1 Department of Clinical Innovative Medicine, Translational Research Center, Kyoto University Graduate School of Medicine, 54 Kawahara-cho, Shogoin, Sakyo-ku, Kyoto 606-8507, Japan

## Abstract

**Background:**

In Japan, only clinical research related to investigational new drug trials must be notified to regulatory bodies, and this lack of a uniform standard for clinical research has caused a number of difficulties. The objective of this study was to assess the willingness of physicians to participate in clinical research and to identify effective methods to promote and enhance clinical research.

**Methods:**

We conducted a cross-sectional survey by administrating questionnaires to physicians in 31 departments in Kyoto University Hospital from October through November 2007.

**Results:**

A total of 51.5% (310 of 602) of physicians completed the questionnaire. More than two-thirds of them reported currently participating in clinical research, and nearly all believed that clinical research is necessary for physicians. Less than 20% of respondents had specific training regarding clinical research, and most reported a need to acquire concepts and skills regarding clinical research, especially those related to statistics. "Paperwork was complicated and onerous" was the most frequently cited obstacle in conducting clinical research, followed by "few eligible patients" and "lack of time". Previous participation in and prospective participation in clinical research, previous writing a research protocol were positively associated with current participation in clinical research.

**Conclusions:**

Physicians in university hospitals need more training regarding clinical research, particularly in biostatistics. They also require administrative assistance. Our findings indicate that the quality of clinical research could be improved if training in clinical research methodology and biostatistics were provided, and if greater assistance in the preparation of study documents requested by the institutional Independent Ethics Committee were available.

## Background

Good Clinical Practice (GCP) should be used for designing, conducting, recording, and reporting trials that involve the participation of human subjects[1]. This guideline should be followed when generating clinical trial data that are intended to be submitted to regulatory authorities. In the United States, many research sites conduct clinical trials in compliance with GCP standards [2], and the European Clinical Trial Directive made GCP mandatory for all clinical drug trials [3].

In Japan, clinical trials of new drugs can be classified into two categories: investigational new drug (IND) application trials, and studies that do not seek marketing approval (non-notified trials). The former are strictly regulated by the Pharmaceutical Affairs Law[4] and by the Ministry of Health, Labour and Welfare (MHLW) Ordinance on GCP[5], which was adopted in Japan in 1997 and is based on the International Conference on Harmonization of Technical Requirements for Registration of Pharmaceuticals for Human Use (ICH) E6 Guidelines[1]. In striking contrast to other countries, Japanese researchers can conduct clinical trials without notifying or applying to the authorities, unless they require new drug approval (NDA). In fact, there is little legal regulation of non-notified trials in Japan. The only guidance provided by the MHLW is Ethical Guidelines for Clinical Studies [6], which was published in 2003 and has no legal implications. The main difficulty in conducting non-notified trials is that the policies of Japanese ethics committees vary by medical institution or hospital. Thus, a trial that is disallowed by one institution might be approved by another, perhaps without sufficient discussion of its ethical or scientific implications.

Before 2003, applications for IND trials were only submitted by the company responsible for manufacturing and marketing the drug. After revision of the Pharmaceutical Affairs Law, an investigator can now initiate and notify the relevant authorities of an IND trial, which involves strict observance of GCP. As of 2007, the number of investigator-initiated IND trials has been very small, and notified by certain university hospitals, including Kyoto University Hospital (KUH), and the Japan Medical Association. However, many non-notified trials, undertaken in observance of the Ethical Guidelines, have been conducted by KUH and other hospitals.

KUH is one of the seven largest university hospitals in Japan, and a total of 348 faculty, 176 senior residents, and 125 junior residents were employed there as of October 2007. Since 2007, it has been one of seven distinguished research sites chosen by the Ministry of Education, Culture, Sports, Science and Technology to participate in the Coordinating Support and Training Program for Translational Research, which seeks to promote quality in clinical research.

The objective of the present study was to investigate both the willingness of physicians to participate in clinical research and their attitudes toward such research. In addition, we aimed to identify methods of support and training that might assist physicians in conducting research. We aimed that our findings may foster future academic clinical research both at KUH and other university hospitals in Japan.

## Methods

### Respondents and survey administration

From October through November 2007 we conducted a cross-sectional survey of 31 departments in KUH. We initially contacted the directors of each of the 34 departments in KUH to explain the study and to ask for their participation in the study. Thirty-one departments consented to participate. The person in charge of each department distributed the study description and questionnaire by hand or by mail to the physicians belonging to the department, and later collected them. Residents, faculty, and doctoral students (physicians) with medical degree were invited to participate. It was not necessary to obtain ethical approval for this survey, as this survey was out of jurisdiction of Ethical Guidelines for Epidemiological Research[7], which shall be applied to studies on etiology of human disease and diagnostic or therapeutic procedures.

### Questionnaire

An initial questionnaire was prepared to gain a better understanding of the current state of clinical research and to guide development of activities at the Translational Research Center, Kyoto University Graduate School of Medicine. To prepare the questionnaire, we modified and added questions to a questionnaire from a similar study conducted in Tokushima University Hospital [8].

The questionnaire inquired about demographic data--including age range and employment status--and attitudes regarding clinical research, clinical research training, and submission of articles on clinical research. Since ICH E6 guidelines for GCP, an international standard for research ethics, is based on and consistent with the principles of the Declaration of Helsinki, we queried the respondents' knowledge of the World Medical Association Declaration of Helsinki [9].

Using multiple-choice questions, respondents were asked about (1) the benefits of conducting clinical research and desired lecture topics on clinical research; (2) the difficulties of conducting research, among physicians who had participated in such research; and (3) the content of reviewers' comments, among physicians who had submitted a clinical research article. The questionnaire was anonymous, and included a separate form to state freely their name or additional opinions for those physicians who wished to collaborate on any further research project.

### Statistical Analysis

Descriptive analysis was used to examine respondents' perception of the benefits and difficulties of clinical research. Answers to multiple-choice questions were summed and listed in order of frequency. The chi-square test was used to compare the age range and the proportion of physicians employed by internal medicine departments (ie, internal medicine, pediatrics, psychiatry and radiology) among respondents with those among both nonrespondents and national physicians.

Bivariate analyses were performed to identify factors that might be associated with current participation in clinical research. We used chi-square tests for categorical variables and *t *test for continuous variables. The continuous variables in this dataset were age range (decade) and knowledge of Helsinki. Correlation analyses were performed to test for multicollinearity between 5 sets of factors we hypothesized might be highly correlated (age range and status, past participation in clinical research and past submission for publication of a manuscript on clinical research, past participation in clinical research and past writing of a research protocol, past participation in clinical research and prospective participation in clinical research, and past submission for publication of a manuscript on clinical research and past writing of a research protocol). Decisions to include factors in the multiple logistic regression analysis were based on the strength of correlated factors (*r *< 0.75) or a *P *value < .05 on bivariate analyses. We performed multiple logistic regression analysis to identify factors that were correlated with participation in clinical research.

A *P *value of less than 0.05 was considered to be statistically significant. Analysis was performed using STAT View (SAS Institute Inc, Cary, NC).

## Results

### Characteristics of respondents

Among 602 physicians from the 31 departments who received the questionnaire, a total of 51.5% (310 of 602) completed the questionnaire. A total of 175 faculty and 58 residents responded; 173 faculty and 243 residents did not respond (*P *< 0.001). As to age range, 47.8% of nonrespondents were aged 20 to 29, 16.8% of nonrespondents were aged 30 to 39, and 24.2% of nonrespondents were 40 to 49. Table 1 provided age range of respondents. There were statistically significant difference between respondents and nonrespondents on age range (*P *< 0.001). The survey respondents were not representative of all physicians at KUH: Faculty was more likely to complete survey than were residents, possibly because many junior residents did not receive the questionnaire. As junior residents rotate through various specialties, some of the person in charge of each department hesitated to distribute the questionnaire to junior residents. A total of 96 faculty and residents employed in internal medicine departments responded to the questionnaire, and 137 faculty and residents in surgical or other departments responded to the questionnaire. There were 164 nonrespondents in internal medicine departments and 252 nonrespondents in surgical or other departments (*P *= 0.657 vs respondents). In comparison, there were 77358 physicians in internal medicine departments and 90969 physicians in surgical or other departments in hospitals in Japan in December 2006 [10] (*P *= 0.146 vs respondents). Respondents did not differ from nonrespondents and national physicians in the proportion of physicians who belonged to internal medicine departments.

**Table 1:** Characteristics of the 304 respondents

Characteristic	Percent*		
	**resident or doctoral student**	**faculty**	**total**
	**(n = 129)**	**(n = 175)**	**(n = 304)**
Age range			
<= 29	15.5	0.6	6.9
30-39	82.2	31.4	53.0
40-49	2.3	53.1	31.6
>= 50	0.0	14.9	8.6
Internal medicine departments	50.4	36.0	42.1
Current participation in clinical research	48.8	82.3	68.1
Past participation in clinical research	53.5	89.1	74.0
Prospective participation in clinical research	61.2	89.7	77.6
Previous training course in clinical research	11.6	18.9	15.8
Do you consider it is necessary for physicians to conduct clinical research?			
yes	96.1	97.1	96.7
Have you ever written research protocol?			
yes	14.0	51.4	35.5
Have you submitted for publication of a manuscript on clinical research?			
yes	24.8	50.9	39.8
Do you know "World Medical Association Declaration of Helsinki Ethical Principles for Medical Research Involving Human Subjects"?			
I know very well	10.9	28.0	20.7
I know to some degree	81.4	68.0	73.7
I don't know	5.4	2.9	3.9

* Percent values were expressed as ratio in respondents of each age range. Percentage may not total 100% due to missing or blank data.

Table 1 lists the respondents' characteristics by status: resident or doctoral student vs faculty. Six respondents with other status or with blank data for status were deleted. Among respondents, 68% of physicians reported current participation in clinical research; 74% reported past participation in clinical research. More faculty than resident or doctoral student reported past participation in, current participation in and prospective participation in clinical research. Most physicians (97%) believed that it is necessary for physicians to conduct clinical research. More than half of faculty had written a research protocol and reported submitting for publication of a manuscript on clinical research, whereas 14% of counterpart had written a research protocol and 25% of counterpart reported submitting for publication of a manuscript on clinical research. However, only 16% had taken a training course in clinical research offered by either the Japan Clinical Oncology Group (9), Kyoto University Graduate School of Medicine (9), other domestic universities and scientific societies (9), or foreign institutions (2). Most physicians (94%) were aware of the World Medical Association Declaration of Helsinki; 4% were not.

### Attitudes

Respondents were queried regarding the benefits of conducting clinical research. Obtaining a better understanding of disease was the most frequently cited benefit, and was mentioned by 255 physicians (47.3%). Enhanced standing in society or the hospital was the second most frequently cited benefit, and was mentioned by 150 physicians (27.8%), followed by obtaining research grants or awards. Eleven respondents (2.0%) felt that there was no benefit (Table 2).

**Table 2:** Attitude towards clinical research

Question	Percent*(%)
What benefits do you think are brought to physicians of conducting clinical research?	
Physicians can obtain a better understanding of disease	47.3
Physicians will enhance standing in society or in hospital	27.8
Physicians will obtain research grants or awards	12.8
Physicians will obtain credits to be board certified doctor	4.1
There is no benefit to physicians	2.0
Which lecture topics related to clinical research are interesting or useful?	
Statistical analysis	25.3
How to write a protocol	20.7
Paperwork and procedures†	13.2
Cost management for clinical research	12.7
Informed consent form to patients	10.5
Compensation	9.2
Medical ethics	8.0
What were the criticisms of reviewers when you submitted for publication a manuscript on clinical research ?	
Statistical analysis	36.9
Selection of patients	21.0
Aim or meaning of research	19.1
Definition of the technical terms	10.2
Ethical problems	5.7
What difficulties did you meet of conducting clinical research?	
The paperwork was complicated and onerous	26.2
Eligible patients were very few	18.9
Lack time	17.6
Too many examinations were scheduled	11.5
There was no benefit to patients	8.6
I could not continue clinical research because of transfer of physicians	6.3
Patients missed appointments	5.4
Patients didn't consent to take placebo	3.6
Doctor-patient relationships were damaged by offering clinical research	0.8

* Percent values were expressed as ratio in total answers. Percentage may not total 100% due to missing or blank data.

†Paperwork and procedures mean production and management of study documents regarding submission to institutional review board and completion of case report form.

Most physicians (93.2%) wanted to attend lectures or seminars on one or more topics related to clinical research. The most frequently cited desired lecture topics were statistical analysis, how to write a protocol, paperwork and procedures (production and management of study documents regarding submission to institutional review board and completion of case report form), and cost management in clinical research (Table 2).

Respondents who had submitted research papers for publication were asked to indicate the criticisms of reviewers. Statistical analysis was the most frequent reviewer criticism, followed by selection of patients, aim or meaning of research, and definition of technical terms (Table 2).

Regarding the difficulties of conducting clinical research, respondents indicated that the "paperwork was complicated and onerous", that there were "few eligible patients", and that the respondents "lack time" (Table 2).

### Factors associated with current participation in clinical research

Age range had moderate correlation with status (r = 0.635), as did past participation in clinical research with prospective participation in clinical research (r = 0.505). Past participation in clinical research had some correlation with past submission for publication of a manuscript on clinical research (r = 0.413), as did past submission for publication of a manuscript on clinical research with past writing of a research protocol (r = 0.311) and past participation in clinical research with past writing of a research protocol (r = 0.282).

In bivariate analyses, current participation had statistically significant correlation with status, age range, past participation in clinical research, prospective participation in clinical research, past submission for publication a manuscript on clinical research, training course in clinical research, past writing a research protocol and knowledge of the World medical Association Declaration of Helsinki. A multivariable logistic regression model was developed including all these correlated factors as variables. Current participation was positively associated with past participation in, prospective participation in clinical research and past writing of a research protocol (Table 3). Age range of 30-39 was negatively associated with current participation in clinical research: Respondents aged 30 to 39 were less than quarter (odds ratio, 0.24; 95% confidence interval, 0.064-0.907) as likely to participate in clinical research currently as respondents aged 20 to 29. There was no association between current participation and either status or previous training course in clinical research.

**Table 3:** Effect of status, age range, and attitudes to current participation in clinical research

Characteristic	Odds ratio (95% CI)	*P *value*
Status		
resident or doctoral student	reference	
faculty	1.416(0.568-3.531)	0.4554
Age range, y		
<=29	reference	
30-39	0.240(0.064-0.907)	0.0353
40-49	0.354(0.069-1.822)	0.2142
>=50	0.218(0.028-1.684)	0.1442
Past participation in clinical research		
yes	5.680(2.40-13.441)	< 0.0001
no	reference	
Prospective participation in clinical research		
yes	5.756(2.508-13.212)	< 0.0001
no	reference	
Previous training course in clinical research		
yes	2.081(0.678-6.389)	0.2002
no	reference	
Previous writing of a research protocol		
yes	2.631(1.130-6.125)	0.0249
no	reference	
Previous submission for publication of a manuscript on clinical research		
yes	1.798(0.815-3.967)	0.1464
no	reference	
Do you know "WORLD MEDICAL ASSOCIATION DECLARATION OF HELSINKI Ethical Principles for Medical Research Involving Human Subjects"?		
I know very well	4.219(0.561-31.728)	0.1619
I know to some degree	2.457(0.413-14.623)	0.3233
I don't know	reference	

* P < 0.05 is considered statistically significant.

The R2 value was 0.378. CI, confidence intervals

## Discussion

In this questionnaire survey of physicians at KUH, most respondents were currently participating in clinical research and felt that clinical research was necessary. As compared to physicians participating in clinical research, smaller proportions of physicians had formal training in clinical research. The majority reported a need to acquire concepts and skills regarding clinical research, especially those related to statistics. Both previous participation in and prospective participation in clinical research were positively associated with current participation in clinical research, suggesting that physicians who were accustomed to clinical research were participating in and would participate in clinical research.

Our findings indicate that the contention that "doctors (in Japan) simply don't want to take part in clinical trials"[11] is a misunderstanding. Indeed, our results indicate that if an adequate trial infrastructure is present, Japanese physicians are eager to conduct clinical research.

KUH is an important research center in Japan, and this likely explains why the rates of participation in and acknowledgement of the importance of clinical research were high among respondents. Studies have reported a wide range in the percentage of physicians participating in clinical research, from 13% to 90% [12-14]. In a questionnaire survey at Tokushima University Hospital[8], 61% of faculty had contributed to IND application trials and 58% of those wanted to participate in IND application trials, whereas in our survey at KUH, 89% of faculty reported past participation in clinical research. The difference in participation rates could be the result of different criteria of clinical research in the questionnaire. As mentioned above, many non-notified trials are carried out at KUH and other hospitals. Perhaps the rate of participation was high because, with the exception of notified trials, physicians in Japan are able to initiate clinical research with only minimal ethical oversight.

In the present study, the difficulties that physicians faced in conducting clinical research are similar to those noted in previous studies [14-16]. Paperwork was cited as a major hurdle, even though the limited number of regulatory obstacles in Japan would be expected to lessen paperwork demands. Perhaps because physicians have a low opinion of the necessity for preparing and managing study documents, they perceive extra paperwork as onerous. Therefore, we suggest that a clinical support center should be available to provide initial advice and support regarding the production and design of documents, thereby establishing good practice. Lack of time was also reported as a major hurdle. Most physicians in university hospitals in Japan are involved in both patient care and research on molecular and cellular biology including experiments with animals. Because researchers could study molecular and cellular biology on a smaller budget than clinical research, which is the evaluation of new treatment involving human subjects, they studied it since it was introduced to Japan. As a result, there are few highly skilled clinical researchers in Japan and opportunities to learn the principles and methodology of clinical research are limited for young Japanese physicians.

Physicians who are familiar with clinical research are able to conduct clinical research more easily than those who are not, as they know the guidelines and laws necessary for conducting clinical research and can use their pre-existing network of experienced research collaborators [17]. In addition, physicians who have completed clinical trials can obtain funding more easily than those who have not; however, they gain no special treatment or financial incentives [11]. As the majority of physicians indicated that obtaining a better understanding of disease was the greatest benefit of conducting clinical research, the pleasure of discovery would appear to have more than repaid them for their efforts.

In our model with respect to current participation in clinical research, the previous training in clinical research was not found to be a significant factor. As various training providers were reported in this questionnaire, the programs and the length of these training courses should be variable. Universities or university hospitals should develop a standardized training program on clinical research that physicians can learn essential knowledge before they initiate such research.

The current study did have some limitations. The most significant of these is that the clinical research referred to in this survey comprised a variety of research types, ranging from epidemiological and observational studies to clinical trials, including IND application trials. Nevertheless, the research support section that serves the university hospital assists with a variety of clinical research designs, and a commonality of needs among physicians was demonstrated in our survey. Another limitation was that the response rate was much higher among faculty than among resident, which may influence the final logistic regression analysis. In addition, this survey took place at a single institution, so the possibility for generalization is limited. However, the difficulties indicated by respondents were quite consistent with those of prior reports. Moreover, an ongoing international collaboration project is attempting to compare the status and attitudes of physicians, and to seek strategies to promote clinical research. The results of this study have contributed much to the refinement and modification of the questionnaire used for the international attitude study. We aim to identify unique and universal problems regarding academic clinical research, and to submit them to academic societies and governing bodies in order to improve the situation. In addition, after completion of our questionnaire survey, Ethical Guidelines for Clinical Studies were just revised and enacted in April 2009. Under the revised guidelines, investigators are now required to register their trials at a public trial registry, to obtain insurance for trial subjects, and to have adequate training in clinical research. Concern for the welfare of trial subjects may have increased, but this may create another barrier to perform clinical research by requesting more paperwork and more funding for insurance for trial subjects.

## Conclusions

Physicians in university hospitals need more administrative assistance and greater knowledge of the principles and techniques of clinical research, especially the concepts of biostatistics. Our results highlight the need for training in clinical research and biostatistics and the necessity for administrative assistance in the production of study documents requested by the institutional Independent Ethics Committee.

## Competing interests

The authors declare that they have no competing interests.

## Authors' contributions

ES conceived the study and participated in the design, management, data analysis, and preparation of the manuscript. TM participated in the study design and in the preparation of the manuscript. MY participated in the study design and participant recruitment. All authors read and approved the final manuscript.

## Pre-publication history

The pre-publication history for this paper can be accessed here:

http://www.biomedcentral.com/1472-6920/9/75/prepub
